# Bilateral Acute Achilles Tendon Rupture Can Be Effectively Treated Non-operatively

**DOI:** 10.7759/cureus.59511

**Published:** 2024-05-02

**Authors:** Efstathios Konstantinou, Theodoros Mylonas, Theofilos Karachalios, Sokratis Varitimidis, Efstratios D Athanaselis

**Affiliations:** 1 Department of Orthopaedic Surgery and Musculoskeletal Trauma, University General Hospital of Larissa, Larissa, GRC; 2 Orthopaedics, University O Thessaly, Larissa, GRC

**Keywords:** achilles tendon rehabilitation, non-operative management, functional outcome, tendon healing, bilateral achilles tendon rupture

## Abstract

Acute rupture of the Achilles tendon (AT) is a common but debilitating injury that requires immediate diagnosis and effective management. Spontaneous bilateral AT rupture is rare; however, it can lead to severe disability for a significant period. This case report presents a 76-year-old patient who suffered a bilateral AT rupture while engaging in a non-strenuous activity. Upon confirmation of the diagnosis by physical examination and radiologic evaluation, conservative treatment was decided due to the presence of numerous comorbidities. A personalized rehabilitation protocol was implemented, allowing weight-bearing activities using Achilles boots at six weeks. Healing of both ATs was confirmed by an MRI at three months. Our case shows that non-operative treatment of these injuries can result in exceptionally favorable outcomes and should not be disregarded. However, thorough patient compliance and surveillance are prerequisites.

## Introduction

The Achilles tendon (AT), a band of fibrous tissue connecting the calf muscles to the calcaneus, is the conjoined tendon of the gastrocnemius and soleus muscles. It is considered to be the largest and strongest tendon of the human body, loading up to 12 times the body weight [1]. Its main blood supply derives from the posterior tibial artery, peroneal artery branches, and smaller arteries arising from the paratenon [2].

The most typical sight of rupture is located 2-6 cm proximal to the tendon's insertion on the calcaneus, where blood supply is relatively poor [2]. The AT is among the tendons that are prone to rupture, with an incidence estimated at 11-37/10^5 ^cases annually in the US, affecting usually 30-50-year-old men participating in sports activities, whereas women usually suffer a rupture during their fifth and sixth decades due to degeneration [3,4]. Risk factors for such an injury are sports and recreational activities, chronic tendonitis, prolonged fluoroquinolone and steroid use, systemic diseases such as diabetes mellitus, and anatomic foot problems [5,6].

However, the occurrence of bilateral spontaneous Achilles tendon rupture is rare, representing a mere 1% of patients sustaining rupture of the Achilles tendon [7], though usually connected with some of the aforementioned predisposing factors [5]. It is a challenging injury, considering that it leads to severe disability for a significant time since it affects both lower limbs. The decision to manage this injury is multifactorial, considering concomitant health problems and the patient's age, demands, and compliance [8].

We present the case of a bilateral, spontaneous, and non-traumatic Achilles tendon rupture treated non-operatively. We aim to highlight the decision-making for conservative treatment of Achilles tendon rupture and its effectiveness regarding the patient's profile.

## Case presentation

A 76-year-old male patient presented to the emergency department due to a sudden inability to walk and support his weight on both lower extremities. He experienced acute and intense pain in both ankles after attempting to stand up from a sitting position. He reported experiencing intermittent mild pain and discomfort on the posterior surface of distal calves for several weeks before, but there was no traumatic occurrence. His medical history included atrial fibrillation, hypertension, prostate hyperplasia, and elevated cholesterol levels, for which he received prescribed medication. He abstained from smoking, but he consumed alcohol in moderation. There was no medical history of rheumatologic or metabolic disease, and the patient declared that he had no recollection of using corticosteroids or quinolones within the past year.

**Figure 1:**
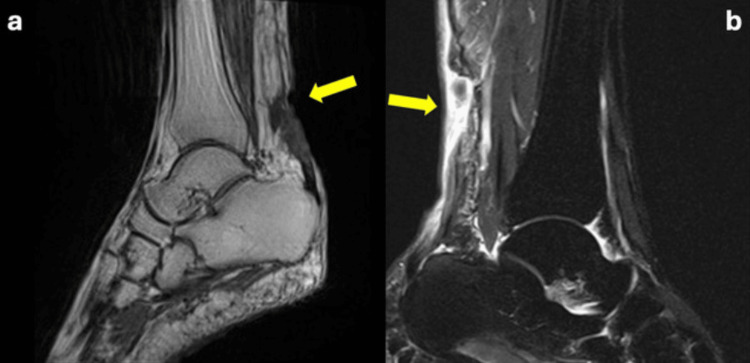
T1-weighted image of the right ankle (a) and T2-weighted image of the left ankle (b) revealing a bilateral Achilles tendon rupture (yellow arrows)

During the clinical examination, the patient was unable to support his weight and execute plantar flexion of both ankles. Bilateral edema and tenderness were observed at the insertion points of the Achilles tendons, along with a palpable gap. He exhibited a positive Thompson's sign in both his lower extremities. No noticeable neurological impairment was observed. Based on the initial radiologic evaluation, there was no evidence of any bony injury. We obtained MRI scans to validate our diagnosis, which confirmed the complete tears of both ATs (Figure 1).

The physical examination was completed after obtaining the Achilles tendon total rupture score (ATRS) [9], which yielded scores of 20 bilaterally. The ankle-hindfoot American Orthopedic Foot and Ankle Society Score (AOFAS) was also determined as 48% for both legs [10].

Concerning the involvement of both legs and the patient's age, the option of non-operative management for both Achilles tendons (ATs) was proposed to the patient, who agreed.

**Figure 2:**
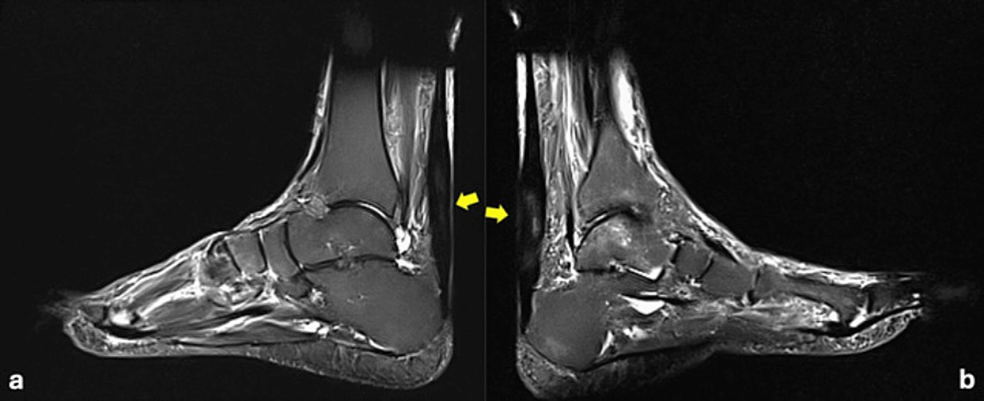
Three-month post-injury MRI of the right (a) and left (b) ankle showing successful healing of Achilles tendons (yellow arrows)

Both ankles were immobilized in below-the-knee braces in an equinus position for four weeks and in a neutral position for the next two weeks. Meanwhile, weight-bearing was not allowed but active motion of knees and toes was encouraged and low-molecular-weight (LMW) heparin was prescribed for six weeks. After the sixth week, ambulation was initiated using Achilles boots in a neutral position to support ankle joints. Protective boots were used for the next six weeks. During this time, daily exercises with active and passive dorsiflexion of both ankles aimed to restore joint flexibility without weight-bearing stress. Unprotected full weight-bearing was permitted at three months after an MRI investigation confirmed the successful healing of both ATs (Figure 2). The rehabilitation process was uneventful.

At the six-month follow-up, the patient could walk normally, and he reported no significant activity limitation. Range of motion (ROM; dorsi- to plantarflexion) was 20-40^o^ for the right ankle and 25-40^o^ for the left. Additionally, the ATRS score was 65, and the AOFAS was 84%. The outcome was evaluated as very satisfying, taking into account the age of the patient.

## Discussion

The presented case indicates the effectiveness and validity of conservative management of Achilles tendon ruptures. Bilateral AT ruptures on an elderly patient with relatively low demands allowed us the option of non-operative treatment, which is a suitable solution for patients who are unfit or unwilling to proceed with operative treatment.

The literature describes and supports both the operative and conservative management of unilateral AT rupture. The advantage of non-operative treatment is that the patient avoids an operation that, even though considered standard, has its technical demands and hazards including risks of anesthesia, superficial or deep trauma infection, re-rupture, and the need for further surgeries [11].

Cases of bilateral AT rupture found in the literature are most commonly traumatic or spontaneous due to chronic wear from repeated stress and prolonged consumption of corticosteroids or quinolones [7,12]. They can pose a significant challenge, as they can impair the ability to walk and perform daily activities, leading to increased disability and decreased quality of life. On the other hand, operative treatment increases risks and costs. Moreover, the management of bilateral acute Achilles tendon rupture differs from that of unilateral rupture, as it requires a more comprehensive approach, close coordination between the patient and the healthcare team, and of course, the patient’s compliance.

Kapoor described a patient with a bilateral Achilles tendon tear, complete on one side and partial on the other [13]. Operative treatment was applied to the first and conservative to the second followed by the same rehabilitation program. The results were comparable with equivalent recovery times from the moment of injury until the resumption of daily activities. Khanzada treated conservatively a spontaneous tendon tear in a 78-year-old female patient with multiple comorbidities who received daily oral quinolones for a urinary tract infection prior to the rupture. Bilateral below-the-knee braces in the equinus position were used for two months with satisfactory functional outcomes at the three-month follow-up [12].

Surgical intervention is typically the preferred approach for treating bilateral Achilles tendon rupture, particularly in younger patients following a traumatic injury [14,15]. Tendon suturing is considered safer and expedites the healing process, according to most surgeons. However, the complications that may arise after operative intervention for a ruptured Achilles tendon must be considered. An increase in risk is associated with bilateral rupture. According to a retrospective study conducted by Stavenuiter, approximately 12% of patients who underwent surgical repair experienced complications such as wound healing complications, re-rupture, and symptomatic venous thromboembolism (VTE) [16]. Advanced age was identified as one of the risk factors associated with these complications.

The satisfying outcome in our patient with bilateral AT rupture due to chronic, degenerative tendon disease, makes us assume that non-operative management could be suitable even for younger patients with healthier and well-vascularized tendons. However, conservative treatment may lead to a prolonged time to return to work [17], although this is not fully supported in the literature [18]. Nonetheless, there is the apparent hazard of non-healing, especially if concerns exist about the patient’s compliance with the rehabilitation program.

Obviously, in case conservative treatment proves ineffective, and the healing process is taking too long, an MRI investigation may be conducted. If an unacceptable gap between the torn edges is still present, the surgical option always remains an alternative, albeit one that requires greater technical expertise. Furthermore, in case a primary repair of the rupture is not possible, surgical techniques, such as flexor hallucis longus (FHL) transfer or V-Y plasty, can be considered suitable alternatives [19].

## Conclusions

A bilateral Achilles tendon rupture is a medical condition that needs to be carefully evaluated and treated since the patient remains non-ambulatory for a significant amount of time. Primary tendon repair is still the most commonly chosen treatment for these cases. Nevertheless, our case suggests that we should not disregard the non-operative treatment of such injuries, as it can yield highly satisfactory outcomes, and the overall recovery time is not considerably longer. Undoubtedly, rigorous patient monitoring and effective cooperation between the patient and medical team are essential.
